# PET/MR imaging of sarcomas: effect of PET quantification by classification of tissue

**DOI:** 10.1186/2197-7364-1-S1-A67

**Published:** 2014-07-29

**Authors:** Claes Nøhr Ladefoged, Adam Espe Hansen, Kim Francis Andersen, Annika Loft, Liselotte Højgaard, Andreas Kjær, Flemming Andersen

**Affiliations:** Dept. of Clinical Physiology, Nuclear Medicine and PET, Rigshospitalet, University of Copenhagen, Kragujevac, Denmark

PET quantification in combined PET/MR can be biased, as the standard Dixon-Water-Fat segmentation (DWFS) attenuation correction (AC) method does not account for bone. Here, we assess PET quantification in PET/MR imaging for patients with bone sarcoma (BS) or soft tissue sarcoma (STS).

We include eight patients (4 BS, 4 STS, 4 MBq/kg [^18^F]FDG) imaged with PET/CT and PET/MR. MR-AC was performed based on: (A) DWFS, (B) DWFS with co-registered and segmented CT bone values superimposed (Bone100), and (C) with co-registered full CT-based attenuation image replacing all voxels within the DWFS body contour (FullCT). We report the mean attenuation value in the sarcoma and the relative difference of a ROI (30% of max isocontour in the sarcoma) activity values for (A)-(B) in reference to (C).

The mean attenuation value in the sarcomas was 2-8% higher in DWFS (μ = 0.1 cm^-1^) compared to FullCT (μ = 0.0926-0.098 cm^-1^) for the voxels not considered bone. The relative difference between PET activity reconstructed using DWFS and FullCT was 5-12% in the STS and insignificant for the BS (max: -2%). Between Bone100 and FullCT we found differences of 10-14% in the STS and 1-9% for the BS.

In sarcomas, PET activity based on MR-AC shows a bias correlated to the location of the sarcoma. However, unexpectedly, the largest error is observed in the STS, which is mainly located away from bone. The error is caused by an overestimation of the attenuation value of soft tissue in DWFS causing an overestimation of the reconstructed PET values. Adding bone further increases the PET values. For the BS these errors roughly cancel each other out. In conclusion, both bone and soft tissue attenuation values differ between DWFS and CT attenuation correction, and contribute to PET quantification errors in sarcomas.

